# Extracellular RNA in Central Nervous System Pathologies

**DOI:** 10.3389/fnmol.2019.00254

**Published:** 2019-10-17

**Authors:** Katharina Tielking, Silvia Fischer, Klaus T. Preissner, Peter Vajkoczy, Ran Xu

**Affiliations:** ^1^Department of Neurosurgery, Charité – Universitätsmedizin Berlin, Berlin, Germany; ^2^Department of Biochemistry, Medical School, Justus Liebig University Giessen, Giessen, Germany

**Keywords:** extracellular RNA, extracellular vesicles, neuroinflammation, CNS pathologies, glioblastoma multiforme, brain metastasis, cerebral occlusive diseases, multiple sclerosis

## Abstract

The discovery of extracellular RNA (exRNA) has shifted our understanding of the role of RNA in complex cellular functions such as cell-to-cell communication and a variety of pathologies. ExRNAs constitute a heterogenous group of RNAs ranging from small (such as microRNAs) and long non-coding to coding RNAs or ribosomal RNAs. ExRNAs can be liberated from cells in a free form or bound to proteins as well as in association with microvesicles (MVs), exosomes, or apoptotic bodies. Their composition and quantity depend heavily on the cellular or non-cellular component, the origin, and the RNA species being investigated; ribosomal RNA provides the majority of exRNA and miRNAs are predominantly associated with exosomes or MVs. Several studies showed that ribosomal exRNA (rexRNA) constitutes a proinflammatory and prothrombotic alarmin. It is released by various cell types upon inflammatory stimulation and by damaged cells undergoing necrosis or apoptosis and contributes to innate immunity responses. This exRNA has the potential to directly promote the release of cytokines such as tumor necrosis factor factor-α (TNF-α) or interleukin-6 from immune cells, thereby leading to a proinflammatory environment and promoting cardiovascular pathologies. The potential role of exRNA in different pathologies of the central nervous system (CNS) has become of increasing interest in recent years. Although various exRNA species including both ribosomal exRNA as well as miRNAs have been associated with CNS pathologies, their precise roles remain to be further elucidated. In this review, the different entities of exRNA and their postulated roles in CNS pathologies including tumors, vascular pathologies and neuroinflammatory diseases will be discussed. Furthermore, the potential role of exRNAs as diagnostic markers for specific CNS diseases will be outlined, as well as possible treatment strategies addressing exRNA inhibition or interference.

## Graphical Abstract

Proinflammatory and prothrombotic extracellular RNA (exRNA) signaling and exRNA in CNS pathologies. **(A)** The interaction of exRNA with the TLR2 agonist Pam2CSK4 results in a synergistic activation of NF-κB signaling, thereby promoting inflammation. The NF-κB pathway is also induced by binding of exRNA to the cell membrane receptor RAGE with consecutive internalization of the nucleic acid. TLR13 which is located on endosomal membranes can also activate NF-κB signaling through interaction with exRNA. TLR3 and TLR7 constitute two more endosomal target receptors of exRNA, which then in turn activate TRIF and IFN-α signaling, respectively. Moreover, exRNA leads to higher levels of IL-6 and TNF- α after binding to TLR8. **(B)** By converting the coagulation factors VII, X, and XII into their active forms, exRNA exerts its procoagulatory function. Furthermore, increased vascular permeability occurs as a consequence of exRNA-VEGF-complexes binding to endothelial VEGF-R2 which subsequently results in disintegration of tight junctions. **(C)** Various exRNAs are associated with pathologies of the central nervous system.

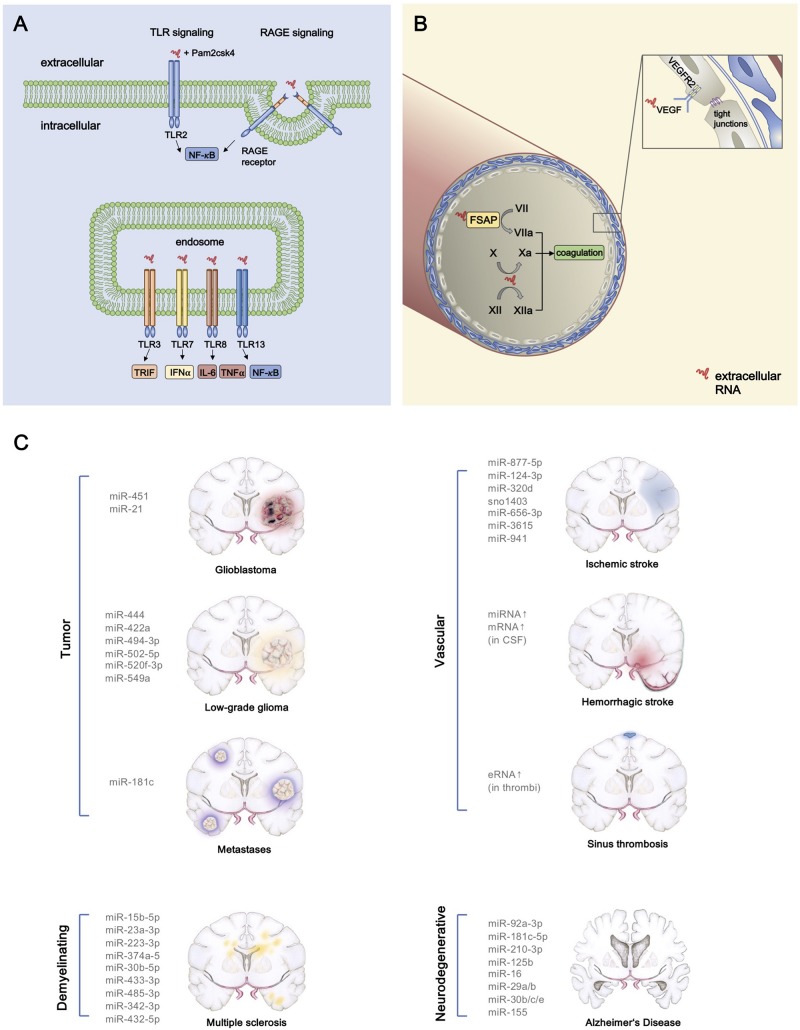

## History of exRna

The discovery of extracellular RNA (exRNA) has shifted our understanding of the role of RNA in complex cellular functions such as cell-to-cell communication, pathology-related biomarkers or as “Danger-associated Molecular Pattern” (DAMP) in the innate immune system. ExRNA was first described in 1928 when Fred Griffith observed that non-pathogenic bacteria obtained pathogenic properties from an infectious, heat-inactivated strain which was later explained by the exchange of nucleic acids (Griffith, 1928; Avery et al., 1944).

The presence of circulating exRNA in human blood was first discovered 1944 (Mandel, 1948). These exRNAs comprise a variety of RNA species which are found outside of cells in which they were transcribed, ranging from small (such as microRNAs) and long non-coding to coding RNAs and ribosomal RNAs. Recent data suggest that different types of exRNA may play a key role in the pathophysiology of various diseases such as myocardial infarction, pulmonary diseases and autoimmune disorders (Lovgren et al., 2004; Ganguly et al., 2009; Cabrera-Fuentes et al., 2014; Biswas et al., 2015; Zimmermann-Geller et al., 2016; Stieger et al., 2017). Current research therefore focuses on its utilization as a diagnostic biomarker and its role as a potential therapeutic target (Saugstad et al., 2017; Stieger et al., 2017).

## Current Knowledge of exRna Regulation

Tumor cells, apoptotic cells and monocytes/macrophages can release RNA into the extracellular space upon inflammatory stimulation. Once secreted or liberated, rexRNAs can act as proinflammatory or prothrombotic alarmins, thereby increasing vascular permeability or acting as prothrombotic cofactors, whereas various miRNAs can be taken up in vesicular form by target cells and alter their genetic program (Fischer et al., 2007; Kannemeier et al., 2007; Bronisz et al., 2014; Fischer et al., 2014). In the absence of inflammation or malignant processes the amount of exRNA in human blood plasma and cell culture supernatants remains low (<100 ng/ml) and increases significantly as a consequence of pathologic processes such as ischemia, infection, apoptosis or necrosis (Fischer et al., 2014). Recent sequencing studies have further characterized the distinct classes of non-cellular exRNAs (Freedman et al., 2016; Danielson et al., 2017; Mick et al., 2017; Umu et al., 2018). The detailed analysis of these exRNAs also poses a technical challenge in the detection, quantification and differentiation of these heterogenous exRNAs. This review sheds light on the characteristics of exRNAs and their potential functional role in CNS pathologies with a focus on vascular diseases, tumor-related, and inflammatory pathologies.

### Diversity of exRNA Species

ExRNAs are a heterogenous group of ribonucleic acids, each of which may have a different impact on the surrounding tissue, either alone or in association with other molecules, such as RNA-binding proteins. The various exRNA subtypes are summarized in Table 1 (Kim et al., 2017). These RNA species vary to a large extent in their abundance and composition, depending both on the investigated cellular compartment as well as the source from which they are extracted such as body fluids, cells, tissues or organs. ExRNAs can be liberated from cells in a free form or bound to proteins as well as in association with MV, exosomes, or apoptotic bodies (Ganguly et al., 2009; Crescitelli et al., 2013). The exact mechanisms of exRNA biogenesis and their vesicular loading have been described elsewhere (Patton et al., 2015; Abels and Breakefield, 2016; Perez-Boza et al., 2018). Analyses of MV-associated exRNAs have shown that miRNAs together with rexRNAs form the majority of the vesicle-associated fraction of exRNA in human blood plasma (Crescitelli et al., 2013; Danielson et al., 2017) In the context of cancer, increased levels of MV-bound extracellular mRNAs have been observed in the blood circulation of patients and in supernatants of malignant tumor cells (Conley et al., 2017; Lazaro-Ibanez et al., 2017; Yokoi et al., 2017).

**TABLE 1:** Types of exRNA.

miRNA	Small, non-coding RNA (21–25 nucleotides) involved in gene regulation (He and Hannon, 2004)
mRNA	Coding RNA that evolves from DNA transcription in the process of protein biosynthesis
tRNA	Small, non-coding RNA (76–90 nucleotides) that translates mRNA sequences in proteins
rRNA	Non-coding, structural component of ribosomes
snRNA	Small, non-coding RNA (about 150 nucleotides) constitutes a part of spliceosomes (Wang et al., 2017)
lncRNA	Long non-coding RNA, >200 nucleotides
circRNA	Small, non-coding RNA contributes to gene regulation by suppressing miRNA function (Wang et al., 2017)
snoRNA	Small, non-coding RNA (60–300 nucleotides) (Wang et al., 2017) modifies tRNA and rRNA chemically (Wang et al., 2017)
piRNA	Small, non-coding RNA (24–32 nucleotides) mainly involved in gene regulation of germ line cells (Wang et al., 2017)
Y RNA	Small non-coding RNA; components of the Ro60 ribonucleoprotein particle, factor for initiation of chromosomal DNA replication (Kowalski and Krude, 2015)

### Established Methods of exRNA Quantification

Methods utilized to detect and quantify exRNA depend on the source of the compounds and the experimental set-up for analysis. Nanoparticle tracking analysis (NTA) and VFC constitute two approaches to analyze the quantity and size of MVs isolated from various sources. The subsequent characterization of a particular exRNA species contained in MVs, exosomes or apoptotic bodies can be conducted by RT-qPCR (Biswas et al., 2015; Saugstad et al., 2017). Analyses of the composition of exRNA are often accomplished by bioanalysis via capillary electrophoresis and RNA sequencing (Fischer et al., 2014; Saugstad et al., 2017). The specific reaction of fluorescent dyes with exRNA provides another (qualitative or semi-quantitative) detection method (Ganguly et al., 2009). Furthermore, dynamics of exRNA-loaded MVs can be monitored by intravital microscopy and immunohistochemistry (van der Vos et al., 2016). In general, the broad heterogeneity of exRNAs combined with the variable cellular fractions and carriers can pose challenges in further analyses and interpretation.

### Functional Properties of exRNA

Although several subtypes of exRNA, including miRNAs and non-coding long RNAs as well as rexRNA, have been described in the context of inflammatory cell signaling, the following subchapter focuses on rexRNA as a direct/indirect extracellular agonist in inflammatory situations.

#### ExRNA as DAMPs and Toll-Like Receptor Ligands

To date, only a few exRNA-dependent cell regulatory mechanisms are known. Receptors belonging to the family of TLR have been shown to be activated by self-exRNA, acting as DAMPs in the immune response toward sterile inflammation or as a result of an infectious stimulus. Ganguly et al. demonstrated that mainly TLR7 and TLR8 play a pivotal role in the recognition of complexes of self-exRNA and the antimicrobial peptide LL37, thereby leading to autoimmune reactions (Ganguly et al., 2009). LL37 is the C-terminal peptide which is proteolytically released from the human cathelicidin protein precursor. It has immunomodulatory properties and prevents the degradation of exRNA and exDNA by forming complexes with (ribo)nucleic acids. Together with exRNA, derived from necrotic cells, LL37 activates MAVS and induces production of IFN-β to support maturation of DC (Zhang et al., 2016). Moreover, the exRNA-LL37 complexes are capable of activating TLR7 in DC, subsequently triggering the secretion of IFN-α, but not Interleukin-6 (IL-6) or Tumor necrosis factor-α (TNF-α).

The activation of TLR8 by the complex can also lead to differentiation of myeloid DC into mature DC and the release of IL-6 and TNF-α. As plasmacytoid and mature myeloid DC accumulate in psoriatic lesions at different stages of the disease, this may indicate that complex formation of exRNA and LL37 initiates the autoimmune response and ensures its preservation (Ganguly et al., 2009). In contrast, no activation of TLR7 (and TLR3) by exRNA alone has been observed in macrophage cell cultures, whereas a synergistic effect of exRNA on TLR2-activation together with its agonist Pam2CSK4 results in the increased expression of cytokines (Noll et al., 2017). Moreover, in the setting of myocardial ischemia, cell-free RNA has been described to augment apoptosis of cardiac muscle cells by activation of TLR3-Trif signaling pathways (Chen et al., 2014). As previously described for TLR7, TLR8, and TLR13, recognition of exRNA can be accomplished by binding to the membrane-bound RAGE resulting in the internalization of the nucleic acids and activation of NF-κB pathways (Kierdorf and Fritz, 2013; Bertheloot et al., 2016).

#### Stimulation of Cellular Expression and Release of TNF-α by RexRNA

The level of rexRNA and TNF-α were found to be increased in human blood plasma during cardiac surgery in the transient perioperative ischemic situation (Cabrera-Fuentes et al., 2014). The same effect has been noted in ischemia/reperfusion models in mice and in isolated rat hearts, whereby cardiomyocytes have been identified as a major source of rexRNA and, to a smaller extent, smooth muscle cells and myofibroblasts. Functionally, exRNA and TNF-α act in a feed-forward loop to promote cardiac reperfusion injury: increase in exRNA leads to an accumulation of TNF-α via activation of TACE, and in turn, TNF-α release will provoke an increase of exRNA as well (Cabrera-Fuentes et al., 2014). TACE, also known as “ADAM metalloproteinase domain 17” (ADAM17), is a metalloproteinase that not only cleaves the cell membrane-bound TNF-α precursor but a variety of more than 50 other cell-bound substrates, including IL-6 receptor, VEGF-receptor 2 or NOTCH (Kanzaki et al., 2016; Li et al., 2018). Both exRNA and TNF-α subsequently induce the expression of inflammatory factors such as iNOS and “Monocyte-chemoattracting Protein” (MCP)-1 to amplify the extent of inflammation (Cabrera-Fuentes et al., 2014). Furthermore, macrophages in cell culture exposed to rexRNA have been shown to undergo a change in cellular characteristics from a so-called anti- (M2) to a proinflammatory (M1) phenotype, resulting in upregulation in gene expression of inflammatory markers such as TNF-α, iNOS, IL-1β, and IL-6 (Cabrera-Fuentes et al., 2015).

Together, these relationships are in favor of a still hypothetical but fundamental type of rexRNA-dependent inflammatory cascade, starting with the exposure of exRNA (as a universal alarmin or DAMP) at any damaged or infected tissue site in the body. The subsequent triggering of proximally located TACE by rexRNA in a cell type specific manner (as described for macrophage TNF-α) will result in the production of inflammatory or other protein products, derived from proteolytic cleavage of the corresponding substrates by TACE. Thus, the “non-specific” alarming factor rexRNA appears to promote site-specific cellular responses, some of which are of profound inflammatory relevance.

#### Influence of RexRNA on Blood Coagulation

It has been demonstrated that certain proteolytic reactions in the intrinsic phase of blood coagulation, termed “contact phase activation” are promoted by polyanionic molecules such as polysaccharides, polyphosphates and rexRNA (Nakazawa et al., 2005; Kannemeier et al., 2007). Here, exRNA can augment activation of the coagulation factors XII and XI by providing a cofactor template for their proteolytic auto-activation (Kannemeier et al., 2007). Thus, targeting rexRNA by RNase1 has been proposed as a novel antithrombotic intervention, as was demonstrated in thrombosis models in mice. Moreover, histidine-rich glycoprotein in plasma can bind to rexRNA (and also to DNA), neutralizing their prothrombotic function and serving as a natural anticoagulant protein (Vu et al., 2015).

Another example of a rexRNA-binding proenzyme is the FSAP, a circulating multifunctional enzymogen, which becomes auto-activated by specific glycosaminoglycans and rexRNA that convert single-chain FSAP into the active two-chain form. Subsequently, active FSAP cleaves/activates several proteins of coagulation and fibrinolysis, but also inactivates inhibitory proteins of coagulation, thereby enhancing the net procoagulant level of the biological system (Nakazawa et al., 2005). In addition, following vascular injury in mice, FSAP exhibits vascular remodeling functions by reducing neointima formation and vascular smooth muscle cell proliferation, leading to a decreased risk of stenosis development (Daniel et al., 2016). Hence, exRNA potentially supports the binary function of FSAP in coagulation and its role in inhibiting neointima proliferation (Nakazawa et al., 2005).

#### Regulation of Vascular Permeability and Infection by RexRNA

Vascular integrity and the adhesion characteristics of inflammatory cells and bacteria can be modulated by rexRNA as well (Fischer et al., 2007, 2012; Zakrzewicz et al., 2016). For example, after exposure to exRNA, cultured capillary BMEC respond with an increased permeability, mediated by VEGF. Thus, high affinity binding of exRNA to VEGF results in the decomposition of endothelial tight junctions and edema formation, initiated by VEGF-receptor 2 activation (Fischer et al., 2007). In cremaster muscle venules, rexRNA was shown to induce leukocyte adhesion and transmigration *in vivo*, and, together with the rexRNA-induced release of proinflammatory cytokines from monocytes, a potent inflammatory response was achieved (Fischer et al., 2012). A recent study also showed that exRNA-containing MV from mast cells promoted an increase in cytokine expression of endothelial cells (Elsemuller et al., 2019). Since rexRNA can avidly bind to particular cell surface attached eukaryotic basic proteins such as extracellular enolase, and also to its bacterial counterpart, host-derived rexRNA serves to promote the adhesion of streptococci to endothelial and epithelial cells (Zakrzewicz et al., 2016). The above findings underline the proinflammatory and vessel-damaging potential of circulating rexRNA.

#### Pharmacological RexRNA Interference

The functional properties of rexRNA, particularly its proinflammatory activities as outlined, are effectively targeted by natural vascular ribonuclease 1 (RNase1), the identical endocrine counterpart to the RNase1 of the exocrine pancreas and TAPI, a TACE inhibitor (Cabrera-Fuentes et al., 2014). Vascular RNase1 is constitutively expressed and secreted by endothelial cells of large and medium blood vessels, but also stored in endothelial Weibel-Palade bodies, from which it can be released by moderate stimulation *in vitro* and *in vivo* (Fischer et al., 2011). By removing the damaging rexRNA, RNase1 can suppress the TNF-α release in hypoxic settings and a reduction of the inflammatory response, or it can decrease the endothelial leakage, thus serving as a vessel- and tissue-protective factor (Fischer et al., 2007; Cabrera-Fuentes et al., 2014). In contrast, the long-term exposure to TNF-α or thrombin can suppress the expression and secretion of endothelial RNase1 (Gansler et al., 2014). RNase1 has also been associated with antimicrobial functions by inhibiting the rexRNA-mediated pneumococcal infection of alveolar epithelial cells (Zakrzewicz et al., 2016). Application of RNase1 has also been discussed as an antitumoral agent; RNase1 administration reduced tumor volume and weight, and increased the area of necrosis *in vivo* in xenograft mice models (Fischer et al., 2013; Zakrzewicz et al., 2016).

Another inflammatory target for rexRNA-induced inflammation is TACE, the sheddase responsible for the release of TNF-α from macrophages. Here, the TACE inhibitor TAPI was shown to inhibit exRNA-mediated shedding of TNF-α in mouse bone marrow-derived macrophages as well as in different *in vivo* models of cardiovascular disease, including cardiac ischemia/reperfusion injury (Cabrera-Fuentes et al., 2014, 2015). In addition, increased adhesion of leukocytes to endothelial cells induced by rexRNA *in vivo* was attenuated by TAPI (Fischer et al., 2012).

## ExRna in Cns Pathologies

Various exRNA species have been investigated in the context of CNS pathologies (Table 2) using *in vivo* and *in vitro* models, with miRNA being the most studied subtype. MiRNAs are small, non-coding nucleic acids and consist of about 22 nucleotides. Released under various stimulatory conditions from any cell type, predominantly in MV-bound form, they are taken up by target cells to modulate their protein expression profile. Together with the Argonaute family of proteins, miRNAs provoke RNA silencing and mRNA degradation by constraining translation, and recruitment of responsible factors leading to mRNA decomposition (Ha and Kim, 2014). Thus, miRNAs serve to transmit cell-to-cell communication on the basis of rearranging the proteome of target cells.

**TABLE 2:** exRNA in CNS pathologies.

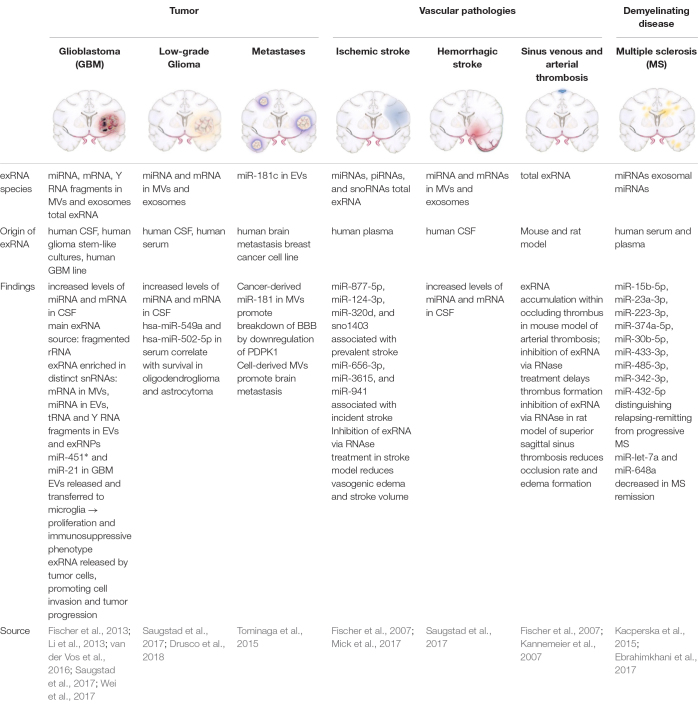

*MVs, microvesicles; CSF, cerebrospinal fluid; RNPs, ribonucleoproteins; miR, microRNA; EVs, extracellular vesicles; BBB, blood brain barrier. ^∗^Might be an artificial effect due to FBS medium.*

### CNS Tumors

#### Glioblastomas

Glioblastomas are high-grade gliomas and represent about 30% of all brain tumors (Ricard et al., 2012). The abundance of studies investigating the role of exRNAs, particularly miRNAs derived by glioblastoma cells, underlines their importance as potential biomarkers and possible therapeutic targets (Dong et al., 2014; Akers et al., 2015; Sathyan et al., 2015; Shi et al., 2015). It has been shown that increased levels of MV-associated miRNAs and also mRNAs are present in the CSF of patients suffering from GBM (Saugstad et al., 2017). In order to capture the entire repertoire of exRNAs in GBM, a recent study has characterized the composition of cancer-derived exRNAs in their association with MV, exosomes, or RNPs, using minimally-biased quantitative analysis (Wei et al., 2017). Each subfraction exhibited a specific RNA-composition with MV-associated exRNAs being the closest to the cellular transcriptome (Wei et al., 2017). Extracellular small RNAs and fragmented rRNA (designated as rexRNA) were demonstrated to form the majority of exRNAs. Tumor-derived mRNA in CSF has been shown to be preferentially entrapped in MVs, whereas miRNAs are primarily associated with exosomes (Wei et al., 2017). In particular, MV-associated miR-451 and miR-21 were shown to be incorporated by murine microglial cells *in vivo* and *in vitro*, subsequently leading to a shift in the phenotype of these cells toward immune suppression (van der Vos et al., 2016). It still remains to be discussed whether the observed increase of miR-451 originates from GBM cells, since this miRNA was also found to be derived from the serum component of the cell culture medium (Tosar et al., 2017). RexRNA may play a crucial role in the context of tumor infiltration and progression as well, since under hypoxic conditions, GBM cells release elevated levels of rexRNA in comparison to non-tumor cells, and after exposure to rexRNA *in vitro*, the adhesion of tumor cells to the endothelium was shown to be increased due to extensive TNF-α release originating from tumor-infiltrating macrophages (Fischer et al., 2013).

#### Low-Grade Glioma

Low-Grade Gliomas represent a heterogenous group of tumors with a low proliferation rate that originate from different glial cells, such as astrocytes, ependymal cells and oligodendrocytes (Louis et al., 2007). Analyses of the exRNA-loaded MVs derived from the CSF of patients with LGG demonstrated higher amounts of mRNA and miRNA as compared to controls (Saugstad et al., 2017). A recent study identified six miRNAs that were overexpressed in tumors of glial origin: miR-4443, miR-422a, miR-494-3p, miR-502-5p, miR-520f-3p, and miR-549a (Drusco et al., 2018). Furthermore, miR-549a and miR-502-5p expression correlated with prognosis in patients with tumors of glial origin (Drusco et al., 2018).

#### Brain Metastases

Cerebral metastases are the most common intracranial tumors, comprising more than 50% of CNS malignancies. The most common primary tumors which metastasize to the CNS are lung, breast, colon, kidney and skin cancer. A recent study in a model of metastatic breast cancer showed that cancer-derived MVs trigger the breakdown of the BBB (Tominaga et al., 2015). Specifically, miR-181c secreted by metastatic cells leads to BBB disruption which in turn supports the delocalization of circulating malignant cells into healthy brain tissue (Tominaga et al., 2015).

### Vascular Pathologies

#### Ischemic Stroke

Ischemic stroke is the leading cause of cerebral strokes due to an occlusion of arteries supplying the brain with blood. A recent study utilized unbiased next-generation sequencing and high-throughput PCR using plasma from 40 participants of the Framingham Heart Study. They identified seven exRNAs (6 miRNAs, 1 snoRNA) that were associated with the incidence or prevalence of stroke (Mick et al., 2017). In rat models of transient focal cerebral ischemia the animals developed vasogenic edema after occlusion, whereas animals that received a pretreatment with RNase1 were protected against edema formation. Moreover, ischemic stroke volumes significantly decreased in pretreated animals, suggesting a neuroprotective function of RNase1 (Fischer et al., 2007). It was further demonstrated that the formation of brain edema was promoted by reduction of the BBB tight junction proteins claudin-5 and ZO-2 which are essential for barrier integrity (Fischer et al., 2007; Jiao et al., 2011; Zhao et al., 2016). Pretreatment of animals with RNase1 led to the preserved localization of these proteins, indicative for the vessel-protective character of the enzyme (Fischer et al., 2007).

#### Hemorrhagic Stroke

Subarachnoid hemorrhage is a form of intracranial bleeding into the subarachnoid space, between the pia mater and arachnoid mater. Inflammatory changes of the brain parenchyma in patients and mice post-SAH have been previously described and were accompanied by an intraparenchymal accumulation of microglia with subsequent neuronal apoptosis (Schneider et al., 2015; Atangana et al., 2016). Patients suffering from SAH demonstrated elevated levels of MV- and exosome-associated miRNA and mRNA in the CSF, underlining the proinflammatory intra- and extracerebral milieu post-SAH (Saugstad et al., 2017).

#### Thrombosis in the Brain

*Sinus sagittalis* thrombosis is a very rare occlusive disease of cerebral sinuses that can be caused by a variety of factors including infections, oral contraceptives, intracranial hypertension, coagulation disorders or neurosurgical operations (Xu et al., 2017; Miao et al., 2018). It has been shown that pretreatment with RNase1 significantly reduced the sinus occlusion rate, comparable to the effect induced by heparin application in rat sinus venous thrombosis models. The development of perivascular edemas was also found to be decreased in pretreated animals. Furthermore, intravenous application of anti-VEGF-antibodies prior to occlusion led to reduced thrombus formation and edema development in the same way as it has been observed after RNase1 treatment (Fischer et al., 2007).

### Multiple Sclerosis

Multiple sclerosis is a demyelinating disease that leads to chronic inflammation of the CNS, most commonly in young adults, and is caused by both environmental and genetic factors (Baecher-Allan et al., 2018). In a study on patients with MS, peripheral blood mononuclear cells and circulating miR-145 were significantly elevated (Sondergaard et al., 2013). Another study showed that miR-648a was significantly reduced in peripheral blood samples of patients in remission compared to healthy individuals (Kacperska et al., 2015). Similarly, expression of miR-let-7a, which exhibits anti-inflammatory properties by inducing IL-10 and IL-13, has been found to be decreased in patients in remission compared to controls (Kacperska et al., 2015). A recent study identified a group of nine serum exosomal miRNAs (miR-15b-5p, miR-23a-3p, miR-223-3p, miR-374a-5p, miR-30b-5p, miR-433-3p, miR-485-3p, miR-342-3p, and miR-432-5p) that may distinguish relapsing-remitting from progressive MS disease (Ebrahimkhani et al., 2017).

### Neurodegenerative Diseases

Neurodegenerative diseases such as AD, ALS, or PD are associated with a progressive loss of CNS functions. Recent studies have focused on the potential of exRNA as diagnostic biomarkers in these diseases. Both, patients with mild cognitive impairment as well as those with AD showed higher plasma or serum levels of miR-92a-3p, miR-181c-5p, miR-210-3p, and miR-125b. An increase of miR-16, miR-29a/b, miR-30b/c/e and miR-155 could be shown in the blood of patients suffering from PD (Margis et al., 2011; Maciotta et al., 2013; Tan et al., 2014; Serafin et al., 2015; Caggiu et al., 2018; Siedlecki-Wullich et al., 2019). ALS and PD have also been associated with the upregulation of specific miRNAs in CSF and blood, although these findings may not always be consistent (Hosaka et al., 2019).

### Perinatal and Traumatic Brain Injury

While specific miRNAs have been directly associated with the consequences of perinatal and traumatic brain injury such as neuroinflammation, arrested oligodendrocyte maturation, neuronal apoptosis and nerve regeneration, further research is needed to elucidate the precise mechanisms of their involvement (Kumar et al., 2017; Pan et al., 2017; Huang et al., 2018; Cho et al., 2019).

## Perspectives in exRna Research

ExRNAs play a key role in inflammatory and coagulatory pathways and are involved in tumor development and progression. Several regulatory mechanisms and signaling pathways of exRNA have been elucidated in various CNS pathologies. Still, the manifold consequences of the accumulation of nucleic acids in the extracellular space as well as the physiological roles of different exRNAs remain to be further investigated. Vascular RNase1 as an exRNA antagonist and TAPI as an inhibitor of TACE may serve as anti-inflammatory and antithrombotic agents with vessel- and neuroprotective properties. To date, the class of miRNAs remains the most well-characterized exRNA species, although they constitute only a fraction of the repertoire of extracellular ribonucleic acids. It is therefore crucial to expand the focus of research toward other exRNA species. The examination of the appearance and localization of exRNA in response to various pathologies as well as the assessment of the mechanisms for exRNA liberation, regulation and function is critical for future utilization as a diagnostic and prognostic biomarker, and as a therapeutic target.

## Author Contributions

KT and RX wrote the manuscript. RX created the figure. SF, KP, and PV revised the manuscript.

## Conflict of Interest

The authors declare that the research was conducted in the absence of any commercial or financial relationships that could be construed as a potential conflict of interest.

## Abbreviations

AD: Alzheimer’s Disease
ADAM17: A disintegrin and metalloproteinase domain 17
ALS: amyotrophic lateral sclerosis
AMP: antimicrobial peptide
BBB: blood brain barrier
BMECs: brain-derived endothelial cells
circRNA: circular ribonucleic acid
CNS: central nervous system
CSF: cerebrospinal fluid
DC: dendritic cell
DNA: desoxyribonucleic acid
EV: extracellular vesicle
exDNA: extracellular desoxyrubonucleic acid
exRNA: extracellular ribonucleic acid
FBS: fetal bovine serum
FSAP: factor VII-activating serine protease
GBM: glioblastoma multiforme
IFN-α: interferon alpha
IFN-β: interferon beta
IL-10: interleukin-10
IL-13: interleukin-13
IL-6: interleukin-6
LDH: lactate dehydrogenase
LGG: low-grade glioma
lncRNA: long non-coding ribonucleic acid
MAVS: mitochondrial antiviral signaling protein
miRNA: micro-Ribonucleic acid
mRNA: messenger ribonucleic acid
MS: multiple sclerosis
MV: microvesicle
NF- κ B: nuclear factor “kappa-light-chain-enhancer” of activated B-cells
NTA: nanoparticle tracking analysis
PD: Parkinson’s Disease
piRNA: Piwi-interacting ribonucleic acid
RAGE: receptor for advanced glycation end-products
rexRNA: ribosomal extracellular ribonucleic acid
RNA: ribonucleic acid
RNase 1: Vascular ribonuclease 1
RNP: ribonucleoprotein
rRNA: ribosomal ribonucleic acid
RT-qPCR: reverse transcription-quantitative polymerase chain reaction
SAH: subarachnoid hemorrhage
snoRNA: small nucleolar ribonucleic acid
snRNA: small nuclear ribonucleic acid
TACE: tumor necrosis factor alpha converting enzyme
TAPI: tumor necrosis factor alpha processing inhibitor
TLR: toll-like receptor
TLR13: toll-like receptor 13
TLR2: toll-like receptor 2
TLR3: toll-like receptor 3
TLR7: toll-like receptor 7
TLR8: toll-like receptor 8
TNF-α: tumor necrosis factor alpha
tRNA: transfer ribonucleic acid
VEGF: vascular endothelial growth factor
VEGF-R2: vascular endothelial growth factor receptor 2
VFC: vesicle flow cytometry
ZO-2: zona-occludens 2.
